# Fluoroalkyl POSS with Dual Functional Groups as a Molecular Filler for Lowering Refractive Indices and Improving Thermomechanical Properties of PMMA

**DOI:** 10.3390/polym10121332

**Published:** 2018-12-02

**Authors:** Kazunari Ueda, Kazuo Tanaka, Yoshiki Chujo

**Affiliations:** 1 Department of Polymer Chemistry, Graduate School of Engineering, Kyoto University, Katsura, Nishikyo-ku, Kyoto 615-8510, Japan; rouge_asteroide_agile_tigre_53@yahoo.co.jp (K.U.); chujo@poly.synchem.kyoto-u.ac.jp (Y.C.); 2 Matsumoto Yushi-Seiyaku Co., Ltd., 2-1-3, Shibukawa-cho, Yao-City, Osaka 581-0075, Japan

**Keywords:** POSS, filler, low refractive material, thermal stability, fluoropolymer

## Abstract

The dual-functionalized polyhedral oligomeric silsesquioxane (POSS) derivatives, which have the seven fluorinated alkanes and the single acrylate ester on the silica cube, were designed as a filler for lowering the refractive index (RI) and improving thermomechanical properties in poly(methyl methacrylate) (PMMA). The desired dual-functionalized POSS fillers were prepared, and because of its high miscibility, homogeneous films were readily obtained, by the casting method, with the mixture solutions containing the modified POSS and the polymers. From optical measurements, it was found that the larger effects of lowering the RIs of the PMMA matrices were observed from the modified POSS than those of the octa-substituted POSS derivatives with the homogeneous substituents. It should be mentioned that the degradation temperatures and the storage moduli were able to be greatly elevated by loading the present POSS fillers. Finally, it was demonstrated that the methacrylate ester-tethered POSS should be the most effective filler for modulating PMMA (Δ*n* = −0.020, ΔTd20 = +53 °C, ΔE’/E’ = +72%).

## 1. Introduction

Organic−inorganic polymer hybrids [1], where both components are homogeneously dispersed in nanometer scales and at molecular level, have been widely known to be a scaffold for producing thermally and mechanically-stable materials with unique functions originating from polymer components. Recently, polyhedral oligomeric silsesquioxane (POSS) has been known to be a promising platform for developing molecular fillers to modulate polymer properties [2,3,4,5,6,7]. It was shown that the connection of luminescent dyes with POSS induced unique thermal and optical behaviors [8,9,10,11,12]. More recently, simply by loading the POSS derivatives onto polymer matrices, various functions, such as thermal stability, mechanical properties and fire resistance, were improved [13,14]. Therefore, much effort has been directed for exploring syntheses of new POSS derivatives having superior functions [15,16,17]. We also proposed that POSS is a versatile “element-block”, which is a functional building block composed of heteroatoms [18,19], for constructing functional polymer hybrids, according to the preprogrammed design [20,21,22]. By mixing POSS derivatives with the polymers in the solution, the hybrid materials can be obtained without troublesome sol−gel methods [23,24,25,26,27], and a significant enhancement on the thermal stability was observed [28,29,30,31,32]. In particular, this concept is valid for reconciling trade-off relationships among material properties, such as durability and optical properties [33,34,35].

Tuning of material properties of low-RI polymers has attracted tremendous attention in the development of modern electronic devices. As an anti-reflection film, low-RI polymers can contribute to the improvement of display resolution by suppressing light scattering, as well as enhancement of device efficiency, by facilitating light extraction [36]. Perfluorinated compounds and multi-pore substances are known to work as a low-RI filler for polymers [37,38,39]. However, the introduction of these fillers into polymers often induces critical decreases in thermal stabilities and mechanical properties of the polymer matrices. In particular, due to the intrinsically poor compatibility of the fluorine groups with polymers, critical phase separation readily occurs, leading to significant decreases in film-formability and durability. Creation of pores inside materials by employing hollow particles is also valid for efficiently lowering the RI values, whereas the critical losses of mechanical properties were still not overcome. Thus, the development of low-RI fillers, without any loss of thermal and mechanical properties, is strongly needed.

We have previously reported the design strategy for a molecular filler which can simultaneously lower RIs and enhance the durability of conventional polymers, by employing POSS that have dual types of functional groups [34,35]. The cyclopentyl substituents at the seven vertices in the silica cube were expected to reduce the RI values by creating lower density regions in the matrix. Losses of thermal and mechanical properties were compensated by a single substituent, by forming strong interaction with polymer chains. Furthermore, it was presumed that miscibility toward polymers was improved because of the lower symmetry of the dual-functionalized POSS than those of the octa-substituted POSS with the homogeneous substituents [34,35,40]. Indeed, desired filler effects on the optical and thermomechanical properties were observed from the polymer hybrids containing the dual-functionalized POSS fillers. However, the degrees of filler effects were still small, especially in the poly(methyl methacrylate) (PMMA) which is the conventional low-RI polymer although validity of this strategy was able to be demonstrated. Therefore, further sophistication should be required for receiving practical fillers, based on the dual-functionalized POSS.

Herein, we demonstrate molecular fillers by employing POSS as a platform for modulating the optical and thermomechanical properties of the PMMA. Series of the dual-functionalized POSS derivatives that had seven fluorinated alkanes and a single functional group, such as methyl methacrylate ester, cyclopentyl, and octadecyl, on the silica cube, were prepared and loaded onto the PMMA film. Initially, it was confirmed that the present POSS fillers had higher miscibility than the octa-substituted POSS, with the homogeneous substituents. In addition, the larger filler effects on the optical and thermomechanical properties were obtained. The mechanism is discussed in this manuscript.

## 2. Experimental Section

### 2.1. General

NMR spectra were measured with a JEOL EX–400 (400 MHz for ^1^H, 100 MHz for ^13^C, and 80 MHz for ^29^Si) spectrometer. Coupling constants (*J* value) are reported in Hertz. MASS spectra were obtained on a Thermo Fisher Scientific EXACTIVE spectrometer, for atmospheric pressure chemical ionization (APCI). Scanning electron microscopy (SEM) images were obtained using a JEOL JSM-5600 operator with an accelerating voltage of 10 kV. UV-vis transmittance spectra were recorded with a SHIMADZU UV-3600 UV-vis-NIR spectrophotometer. The refractive indices were determined with an Abbe refractometer DR-M4 (accuracy ±0.0002, ATAGO Co., ltd., Tokyo, Japan) at 580 nm at 25 °C. Dynamic mechanical analysis (DMA) was performed on a SDM5600/DMS210 (Seiko Instrument, Inc. (SII), Tokyo, Japan) with a heating rate of 2 °C/min at 1 Hz, with 1% strain, under air. Differential scanning calorimetry (DSC) thermograms were carried out on an SII DSC 6220 instrument, SII, by using ~10 mg of exactly weighed samples, at a heating rate of 10 °C/min, under nitrogen. The glass transition temperatures (*T*_g_) were evaluated from the second monitoring curves, after annealing at 100 °C for 10 min, followed by cooling to 30 °C. Thermogravimetric analysis (TGA) was performed on an EXSTAR TG/DTA 6220, SII, with a heating rate of 10 °C/min up to 500 °C, under nitrogen atmosphere. Residual chloroform was removed by keeping it in a vacuum oven, at 100 °C for 1 h before the TGA measurements. The van der Waals volumes of the POSS derivatives and the monomer unit of the PMMA were calculated after the modeling, using a semi-empirical AM1 method.

### 2.2. Materials 

Trimethoxy(3,3,3-trifluoropropyl)silane (Tokyo Chemical Industry Co., Ltd., Tokyo, Japan), 3-(trichlorosilyl)propyl methacrylate (Sigma-Aldrich Co. LLC, St. Louis, MO, USA), trichlorocyclopentylsilane (Adrich), trichloro(octadecyl)silane (Adrich), and sodium hydroxide (FUJIFILM Wako Pure Chemical Corporation, Osaka, Japan) were purchased and used for the assays, without further purification. PMMA (*M_w_* = 800,000) was purchased from the Nacalai Tesque (Kyoto, Japan). Tetrahydrofuran (THF) and triethylamine were purchased from FUJIFILM Wako Pure Chemical Corporation and Kanto Chemical Co., Inc., Tokyo, Japan and were purified using a two-column solid-state purification system (Glasscontour System, Joerg Meyer, Irvine, CA, USA).

### 2.3. Syntheses and Preparation of Materials

Synthesis of the Silsesquioxane Partial Cage [Na_3_O_12_Si_7_(C_3_H_4_F_3_)_7_]. Na_3_O_12_Si_7_(C_3_H_4_F_3_)_7_ was synthesized by the following method. Trimethoxy(3,3,3-trifluoropropyl)silane (5.00 g, 22.9 mmol), THF (25 mL), deionized water (0.53 g, 29.4 mmol), and sodium hydroxide (0.40 g, 10.0 mmol) were charged in a round-bottomed flask, equipped with a reflux condenser. After being refluxed for 5 h, the mixture was cooled down to the room temperature and held, with vigorous stirring, for 15 h. The solvent was removed by a rotary evaporator and a white solid was obtained. After being dried in vacuo, 3.6 g of the products were obtained with a 97% yield.

Synthesis of the POSS fillers. Na_3_O_12_Si_7_(C_3_H_4_F_3_)_7_ (2.1 g, 1.8 mmol) and triethylamine (0.27 mL, 2.0 mmol) were dissolved in THF (40 mL) and cooled in an ice bath. Then trichlorosilane (3-methacryloxypropyl-, 0.53 g, 2.0 mmol; cyclopentyl-, 0.41 g, 2.0 mmol; and octadecyl-, 0.79 g, 2.0 mmol) in THF (4 mL) was added slowly to the mixture. The resulting solution was stirred at 0 °C for 4 h and then overnight, at room temperature. After removing the insoluble salts by filtration, the solvent was removed by a rotary evaporator and a white solid was obtained. The white solid was washed with methanol and dried in vacuo to yield the desired product as a white powder (F+MMA POSS, 51%; F+CP POSS, 53%; F+C18 POSS, 54%).

F+MMA POSS: ^1^H NMR (CDCl_3_, 400 MHz) δ 6.10 (s, 1 H), 5.57 (s, 1H), 4.13 (t, 2H, *J* = 6.7 Hz), 2.13 (m, 14H), 1.94 (s, 3H), 1.77 (m, 2H), 0.94 (m, 14H), 0.76 (t, 2H); ^13^C NMR (CDCl_3_, 100 MHz) δ 167.4, 136.6, 127.2 (q, *J* = 276 Hz), 125.4, 66.0, 27.8 (qd, 31 Hz, 4.1Hz), 22.1, 18.2, 7.8, 4.1; ^29^Si NMR (CDCl_3_, 80 MHz) δ –66.0, –67.5, −67.7; HRMS (APCI) [(M + Cl)^−^] calcd. 1256.9853, found 1256.9864.

F+CP POSS: ^1^H NMR (CDCl_3_, 400 MHz) δ 2.14 (m, 14H), 1.80 (br, 2H), 1.69−1.37 (br, 6H), 1.14−0.80 (br, 15H); ^13^C NMR (CDCl_3_, 100 MHz) δ 127.1 (q, *J* = 267 Hz), 27.7 (qd, 31 Hz, *J* = 4.0 Hz), 27.2, 26.9, 21.5, 4.0; ^29^Si NMR (CDCl_3_, 80 MHz) δ –65.7, –67.5, −67.7; HRMS (APCI) [(M + Cl)^−^] calcd. 1198.9798, found 1198.9794.

F+C18 POSS: ^1^H NMR (CDCl_3_, 400 MHz) δ 2.11 (m, 14H), 1.57 (br, 32H), 0.89 (br, 17H), 0.63 (br, 2H); ^13^C NMR (CDCl_3_, 100 MHz) δ 127.2 (q, *J* = 276 Hz), 32.9, 32.1, 29.9, 29.5, 27.9, 22.8, 14.1, 4.11; ^29^Si NMR (CDCl_3_, 80 MHz) δ –65.3, –67.5, −67.8; HRMS (APCI) [(M+Cl)^−^] calcd. 1383.1989, found 1383.2002.

Preparation of the Polymer Composites. The mixtures (20 mL) containing 1 g of PMMA and various amounts of POSS fillers in chloroform, were stirred at room temperature for 3 h and then poured into the bottom of the vessels (7 cm × 4.5 cm). After drying at room temperature for 2 days, the film samples were dried again, in a vacuum oven, at 60 °C for 2 h. The resulting films were used for the following measurements.

### 2.4. Analyses

Determination of the Refractive Indices of the Polymer Composites. According to the Lorentz–Lorenz equation, the refractive index (n) of the polymer (element 1) composites containing fillers (element 2) can be described using the molar fractions (*α*), molar refractions (*R*), and the molar volumes (*V*) [41,42].

(1) $$\frac{n^{2}-1}{n^{2}+2}=\alpha_{1}\frac{n_{1}{}^{2}-1}{n_{1}{}^{2}+2}+\alpha_{2}\frac{n_{2}{}^{2}-1}{n_{2}{}^{2}+2}=\alpha_{1}\frac{R_{1}}{V_{1}}+\alpha_{2}\frac{R_{2}}{V_{2}}$$

The degree of packing can be described by the molecular packing coefficient *K*_p_ that is defined as

(2) $$K_{p}=\frac{V_{VDW}}{V_{int}}$$

where *V*_int_ and *V*_VDE_ are the intrinsic and van der Waals volumes of the molecules, respectively. Therefore, molecules that have significant abilities to lower the interactions between polymer chains would show a smaller *K*_p_ value. In addition, according to the Vuks equation, Equation (1) can be transformed in the Equation (3).

(3) $$\frac{n^{2}-1}{n^{2}+2}=\alpha_{1}\frac{n_{1}{}^{2}-1}{n_{1}{}^{2}+2}+\alpha_{2}\frac{n_{2}{}^{2}-1}{n_{2}{}^{2}+2}=\alpha_{1}\frac{K_{p1}R_{1}}{V_{\mathrm{VDW},1}}+\alpha_{2}\frac{K_{p2}R_{2}}{V_{\mathrm{VDW},2}}$$

In the case of amorphous polymers, the *K*_p_ values (*K*_p1_) were determined to be 0.677 [29]. Therefore, we can simply calculate *K*_p2_ values by measuring the refractive indices of the composite (n), using Equation (3).

## 3. Results and Discussion

**Scheme 1:**
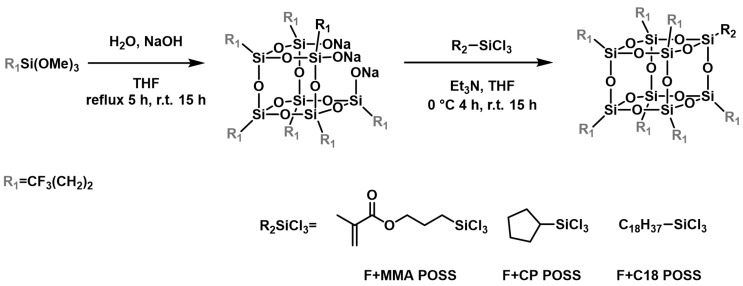
Synthesis of the polyhedral oligomeric silsesquioxane (POSS) fillers that had a dual type of substituents.

To meet opposite demands, the modified POSS derivatives that had the fluoroalkyl and other functional groups, were designed (Scheme 1). According to previous studies, introduction of the fluoroalkyl groups can efficiently lower the RI values of polymers. However, because of the intrinsic very weak interaction with the polymer chains, critical low miscibility of the polymer matrices was presumed. To compensate for the decrease in miscibility, another functional group, which can be expected to form a strong interaction or a bond with the polymer chains, was introduced into the POSS. It was reported that the methacrylate can form a covalent bond with the PMMA, by heating [40]. As a result, reinforcement of the mechanical properties of the PMMA film was capable. Therefore, the methacrylate ester-tethered POSS (F+MMA POSS) was designed. The octadecyl group in the F+C18 POSS was also expected to form strong interaction with PMMA, by alkyl-chain entanglement [23,35]. The cyclopentyl group was introduced as a comparison to the previous dual-functionalized POSS, which can greatly reduce the RI value of the PMMA (F+CP POSS) [35]. The series of the modified POSS derivatives were conducted, according to Scheme 1. The syntheses of the target POSS derivatives were performed via the preparation of the incomplete cage, followed by the formation of the POSS cage, following the procedures reported elsewhere [35,40]. From the NMR and MS measurements, it was confirmed that the products had the desired structures. All POSS derivatives showed good solubility in chloroform, and film samples for optical and mechanical measurements were prepared through casting with the mixture solutions containing each POSS filler and PMMA, followed by pre-heating at 60 °C for 2 h, to remove the solvent. In the case of the F+MMA POSS, the cross-linking reaction could proceed in this step [40].

**Figure 1:**
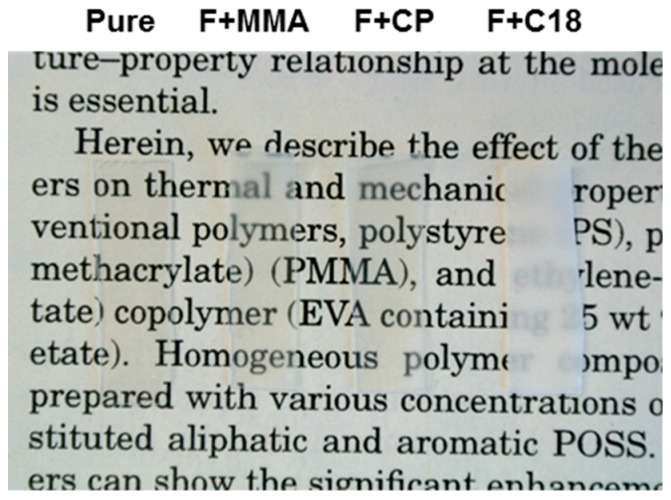
Appearances of the poly(methyl methacrylate) (PMMA) hybrid films with 2 mol% POSS fillers.

The PMMA films containing 2 mol% (ca. 20 wt%) F+MMA POSS and F+CP POSS mixtures had good transparency, whereas white opaque films were obtained from the F+C18 POSS (Figure 1). From the absorption measurements in the wavelength range from 380 nm to 780 nm, the transparency of the film samples was quantitatively calculated as an averaged value in this wavelength region (Table 1 and Figure S1). The polymer films with F+MMA POSS and F+CP POSS mixtures below 2 mol% presented more than 85% transparencies, while the critical decrease in transparency was detected from the hybrids with the F+C18 POSS. These data indicate that miscibility can be improved by introducing another substituent into the POSS core. The surface morphologies of the hybrid films were investigated using SEM. Critical phase separation or aggregation of the POSS fillers in the size range where the RI values were influenced, was slightly observed in the PMMA hybrids with 2 mol% F+MMA POSS and F+CP POSS (Figure S2). Conversely, by adding the same amounts of F+C18 POSS, SEM images with inhomogeneity were obtained. These results indicate that homogeneous dispersions of the F+MMA POSS and the F+CP POSS mixtures were realized in the PMMA. Especially, the F+PMA POSS mixture should have a higher miscibility with the PMMA than the F+CP POSS. It is likely that 3-methacryloxypropyl-group could interact with the polymer chains, leading to high dispersion in the polymer matrices. In the case of commodity polymers, such as polystyrene and PMMA, the turbidity appeared only by adding 1 mol% (ca. 10 wt%) of the previous molecules, such as the octa-substituted POSS fillers with the homogeneous substituents [23,24]. This fact means that the miscibility of the PMMS could be improved. Decreased molecular symmetry by introducing a single functional group could contribute to miscibility improvement by suppressing crystallization. Most samples showed turbidity at 2 mol%, and the transparency of the films decreased. F+C18 POSS induced white opaque into the film, even at 0.5 mol%. Thereby, we discuss the filler effects with the data sets obtained from samples containing 2 mol% F+MMA POSS and F+CP POSS.

**Figure 2:**
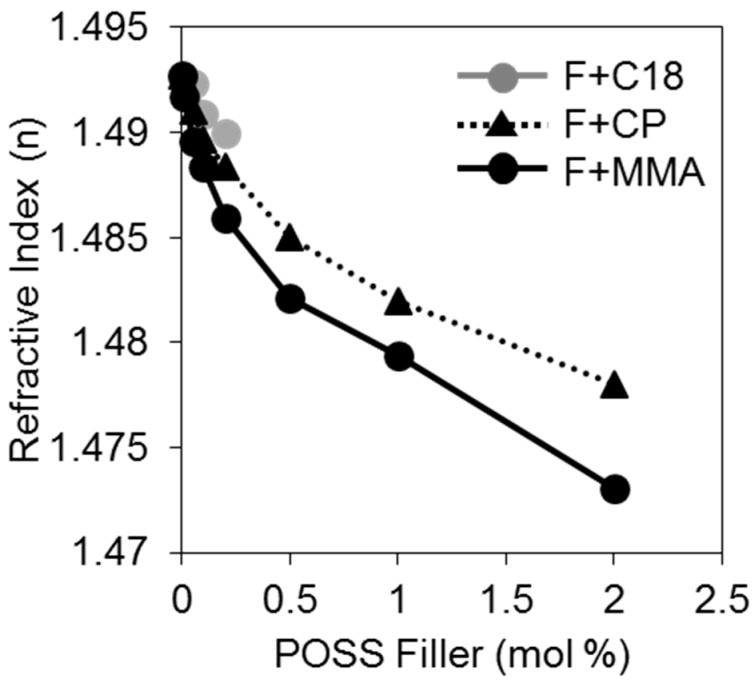
Refractive indices (n) of the PMMA hybrids containing various concentrations of the POSS fillers.

To evaluate the influence of the POSS fillers on the RI values of the PMMA, optical measurements were performed with the film samples containing variable concentrations of the POSS fillers, using an Abbe refractometer (Figure 2). The averages of the values at five distinct points in the films are shown in Table 1. The RI values of the polymer films were drastically lowered by the fluorinated POSS fillers. By loading the F+MMA POSS with 2 mol%, the degree of the reduction was, approximately, 0.02. F+CP POSS filler could also reduce the RI values (2 mol%, Δ*n* = ca. −0.015). It should be mentioned that degree of the lowering effect of the F+CP POSS was much larger than that of the previous dual-functionalized POSS with cyclopentyl groups (ca. −0.005). These data clearly indicated that the fluorinated POSS can have a superior ability to be an efficient filler, for lowering the refractive indices of the PMMA. It is suggested that the POSS mixtures could be completely dispersed in the polymer matrices, by suppressing the crystallinity of the POSS, as mentioned above. Therefore, a lower density region should be efficiently created around the POSS filler, leading to the lowering effects on the refractive indices. According to previous reports, the degree of decrease in RIs, by the present POSS fillers, is large enough for applications in optical fibers [43,44]. Thus, our concept for the design of molecular fillers might be applicable in the practical light-guiding materials.

To estimate the degree of packing in polymer chains and filler molecules, the packing coefficients were calculated according to previous reports (see the Supporting Information) [41,42]. In the absence of the POSS filler, the packing coefficient (*K*_p1_) of the pristine PMMA film was determined as 0.677 [29]. If the POSS filler could create lower density regions in the matrices, a smaller *K*_p2_ value than the *K*_p1_ would be obtained (Table 1) [24]. Apparently, the *K*_p2_ values were smaller than that of the pure PMMA film. These results clearly indicated that the lowering effect on the RI values of the PMMA, by the POSS filler, should originate from the decreases in local density, around the POSS fillers.

Thermal stabilities of the polymer films containing fluorinated POSS fillers were examined by the TGA. Figure S3 shows the TGA curves from the samples containing the POSS fillers with variable amounts, and Table 1 summarizes the *T*_d20_, using the polymer composites containing the POSS fillers. From the curves, it was indicated that degradation occurred through three steps. It was reported that pyrolysis of PMMA proceeds, initially, at the head-to-head linkage, followed by the chain-ends, and the random scission in the main-chains [45]. According to the previous reports, POSS fillers can effectively suppress the second and third steps, which can be evaluated as the degradation temperature with 20% weight losses. Therefore, the thermal stabilizing effects by the POSS fillers were discussed by comparing the *T*_d20_ values of the polymer matrices [28,29,34,40]. Pure PMMA exhibited a *T*_d20_ at 215 °C, and significant thermal reinforcement of the PMMA matrices by loading the POSS fillers can be observed. By increasing the amount of the POSS, the *T*_d20_ values increased. Finally, the *T*_d20_ value of the PMMA matrices containing 2 mol% of the F+MMA POSS and the F+CP POSS increased by 53 and 11 °C, respectively. Comparing to the previous dual-functionalized POSS (Δ*T*_d20_ = +2 °C), larger stabilization effects were obtained. According to the previous reports, the methacrylate ester group connected to the POSS reacted with the PMMA matrices, by heating around 60 °C [40]. As a result, significant enhancement of thermal stability was observed. From the analyses, it was shown that cross-linking between the methacrylate ester moiety and PMMA should occur, followed by the formation of the tight binding of the silica cube to polymer chains. In the current case, it has also been suggested that the drastic thermal reinforcement in the hybrid could be induced by the cross-linking reaction at the methacrylate ester moiety, in the F+MMA POSS.

According to the previous DSC result with the octa-substituted POSS with a single kind of alkyl-substituents [28], *T*_g_ values were slightly influenced by loading the POSS onto PMMA, with high homogeneity. Meanwhile, significant decreases in *T*_g_s were observed in the heterogeneous films (Table 1). Aggregation of the POSS was obviously observed in the F+C18 POSS-containing film. Due to the low miscibility of the F+C18 POSS, relatively lower *T*_g_ should be induced by the filler addition.

Finally, the mechanical properties of the polymer matrices containing the POSS fillers were evaluated by DMA (temperature scan at 1 Hz). The mechanical properties of the polymer composites containing the POSS fillers are listed in Table 1. It was shown that the POSS fillers efficiently improved the rigidity of the PMMA matrices, as represented in the storage modulus (*E’*), although each POSS molecule created a lower density region. The *E’* value was enhanced by 72% with the F+PMA POSS and 71% with the F+CP POSS. In the previous reports, loss of *E*’ was induced with the POSS filler possessing the hepta-substituted cyclopentyl and single fluoroalkyl groups [35]. Molecular motions should be effectively suppressed by the rigid POSS core via the single substituent. From these data involving the DSC, the TGA, and the DMA, it can be summarized that the POSS fillers used in this study have significant abilities to simultaneously improve the thermomechanical properties and lower the refractive indices of polymers.

## 4. Conclusions

By utilizing the molecular fillers based on fluoroalkyl POSS having dual types of functional groups, the polymer hybrids were prepared. From the series of measurements with the hybrid films, it was clearly shown that the POSS fillers can effectively lower the RI values of the PMMA and enhance both the thermal and the mechanical durability. It is proposed that the one functional group should make a strong interaction with the polymer chains, even in the presence of low-miscible hepta-substituents of the fluoroalkyl groups, followed by suppressing the molecular motions. These results indicate that the dual types of functional groups on the POSS core can share roles in simultaneously improving the thermomechanical properties and lowering the refractive indices of the conventional polymers. In other words, it was demonstrated that the POSS played a role only in introducing the positive effects of each functional group, followed by reconciling the trade-off relationship in the polymer functions. Furthermore, it can be said that our concept of designable hybrids, utilizing the preprogrammed-modified POSS could include a huge potential for receiving multi-functional polymeric materials with high stability.

## Figures and Tables

**Table 1:** Summary of the filler effects on the various properties of the PMMA, by the POSS fillers*^a^*.

POSS filler	Transmittance (%) *^b^*	∆n *^c^*	*K*_p2_	*T*_d20_ (°C) *^d^*	Δ*T*_d20_ (°C)	*T*_g_ (°C)	*E*’ (MPa) *^e^*
none	92	−	−	215	−	60.6	1780
F+MMA	87	−0.0197	0.093	268	+53	63.8	3060
F+CP	85	−0.0147	0.180	225	+10	61.8	3050
F+C18	45	−*^f^*	− *^f^*	272	+57	58.6	2630

*^a^* 2.0 mol% POSS. *^b^* Calculated as an averaged value from 380 to 780 nm. *^c^* Averaged value measured at five points. Calculated from the subtraction of the PMMA films from the refractive index (1.4927). *^d^* Determined as the decomposition temperature with 20 wt% weight losses. *^e^* Represented at 30 °C, and the errors are within 5%. *^f^* Not detectable due to being opaque.

## Supplementary Materials

The following are available online at http://www.mdpi.com/2073-4360/10/12/1332/s1.
