# Supplementary material for: Fluoroalkyl POSS with Dual Functional Groups as a Molecular Filler for Lowering Refractive Indices and Improving Thermomechanical Properties of PMMA
Source: Polymers (Basel). 2018 Dec 2;10(12):1332. doi: 10.3390/polym10121332 (PMC6401951; doi:10.3390/polym10121332)
Supplement: Supplementary file 1 [file polymers-10-01332-s001.pdf]

Supporting Information

Fluoroalkyl POSS with Dual Functional Groups as a Molecular Filler for Lowering Refractive Indices and Improving Thermomechanical Properties of PMMA

Kazunari Ueda[1,2], Kazuo Tanaka[1]* and Yoshiki Chujo[1]

[1]Department of Polymer Chemistry, Graduate School of Engineering, Kyoto University, Katsura, Nishikyo-ku, Kyoto 615-8510, Japan

[2]Matsumoto Yushi-Seiyaku Co., Ltd., 2-1-3, Shibukawa-cho, Yao-City, Osaka 581-0075, Japan

Phone: +81-75-383-2604

Fax: +81-75-383-2605

*To whom correspondence should be addressed: tanaka@poly.synchem.kyoto-u.ac.jp

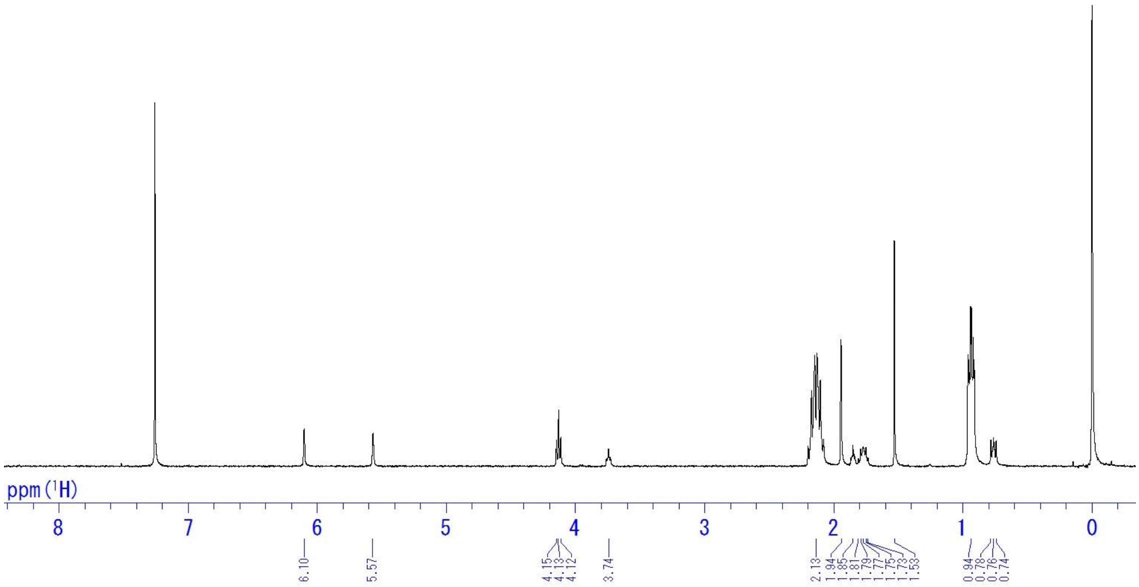

Chart S1. ^{1}H NMR spectrum of F+MMA POSS in $CDCl_3$.

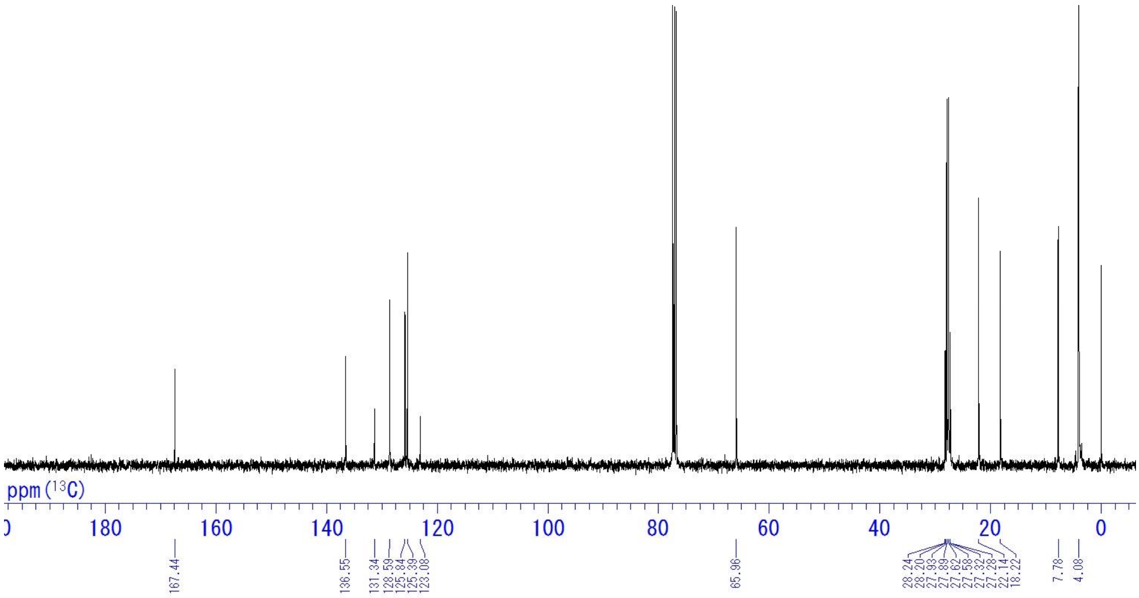

Chart S2. ^{13}C NMR spectrum of F+MMA POSS in $CDCl_3$.

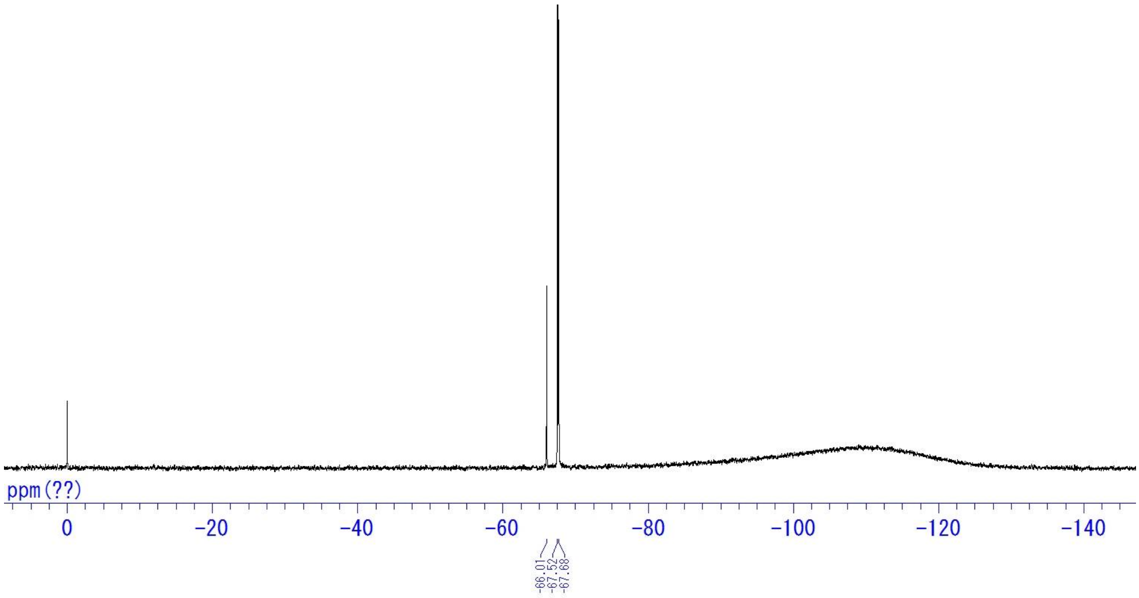

Chart S3. ^{29}Si NMR spectrum of F+MMA POSS in $CDCl_3$.

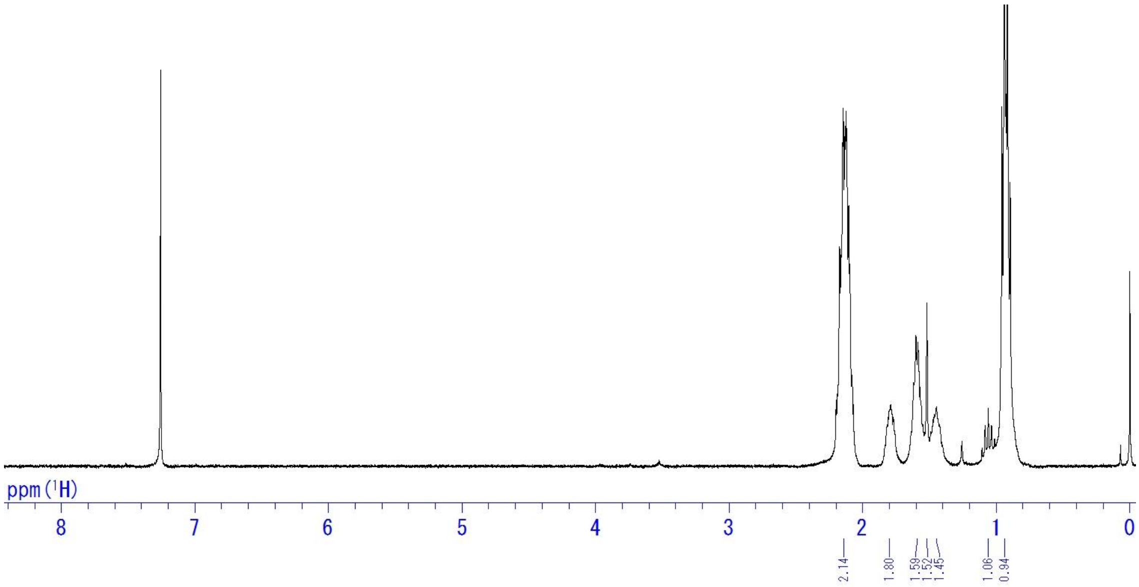

Chart S4. ^{1}H NMR spectrum of F+CP POSS in $CDCl_3$.

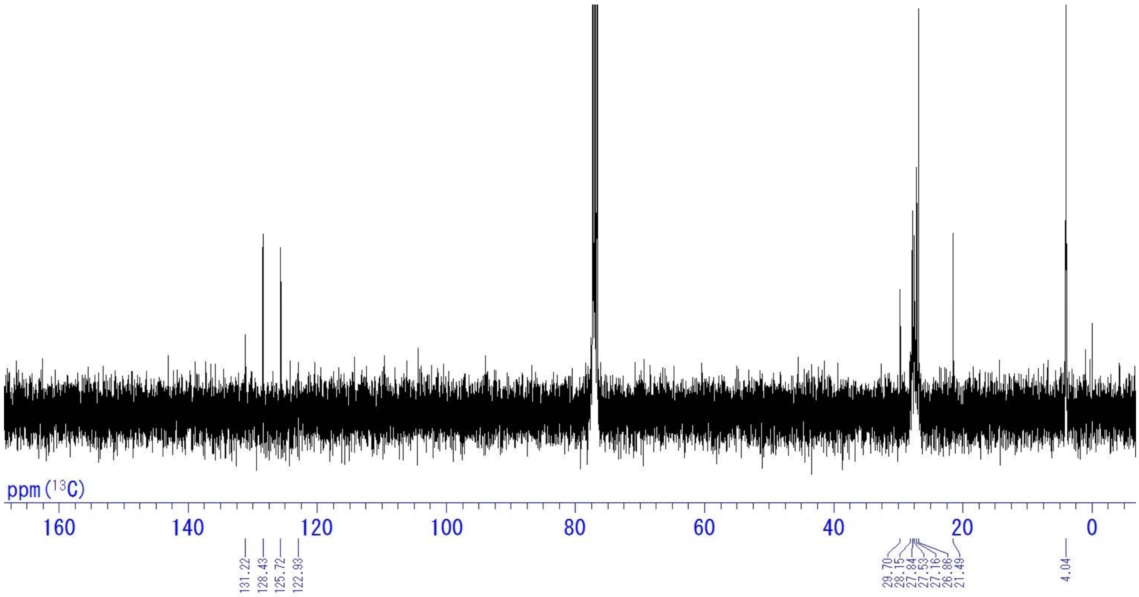

Chart S5. ^{13}C NMR spectrum of F+CP POSS in $CDCl_3$.

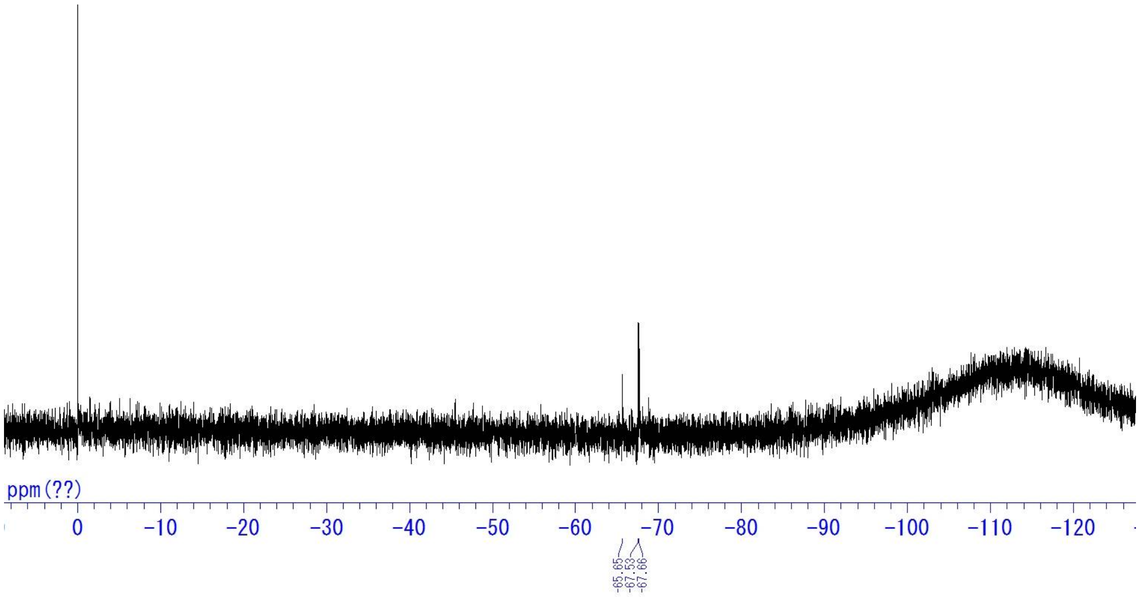

Chart S6. ^{29}Si NMR spectrum of F+CP POSS in $CDCl_3$.

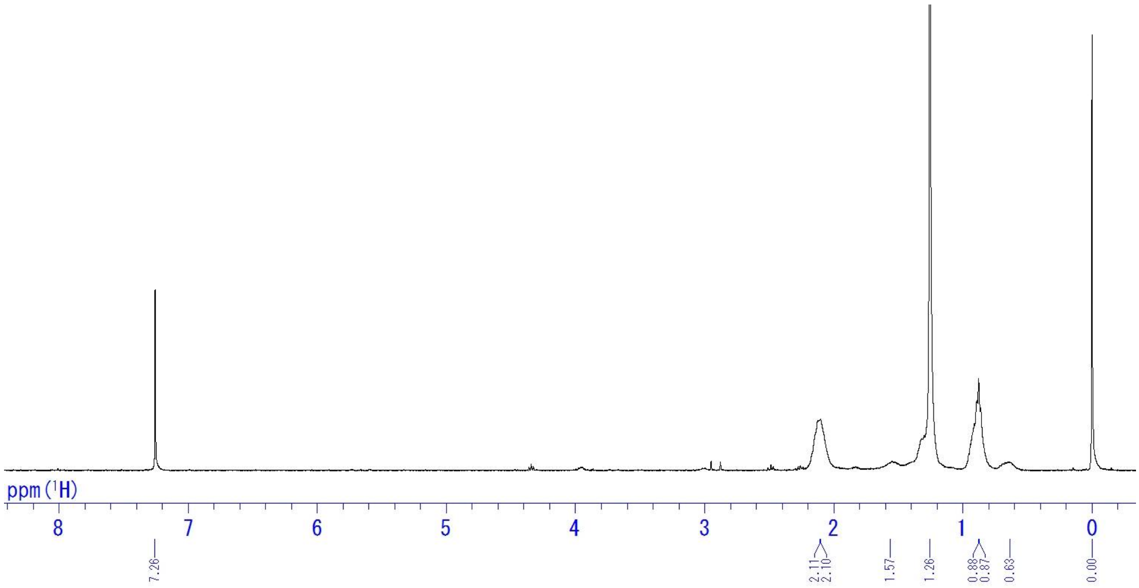

Chart S7. ^{1}H NMR spectrum of F+C18 POSS in $CDCl_3$.

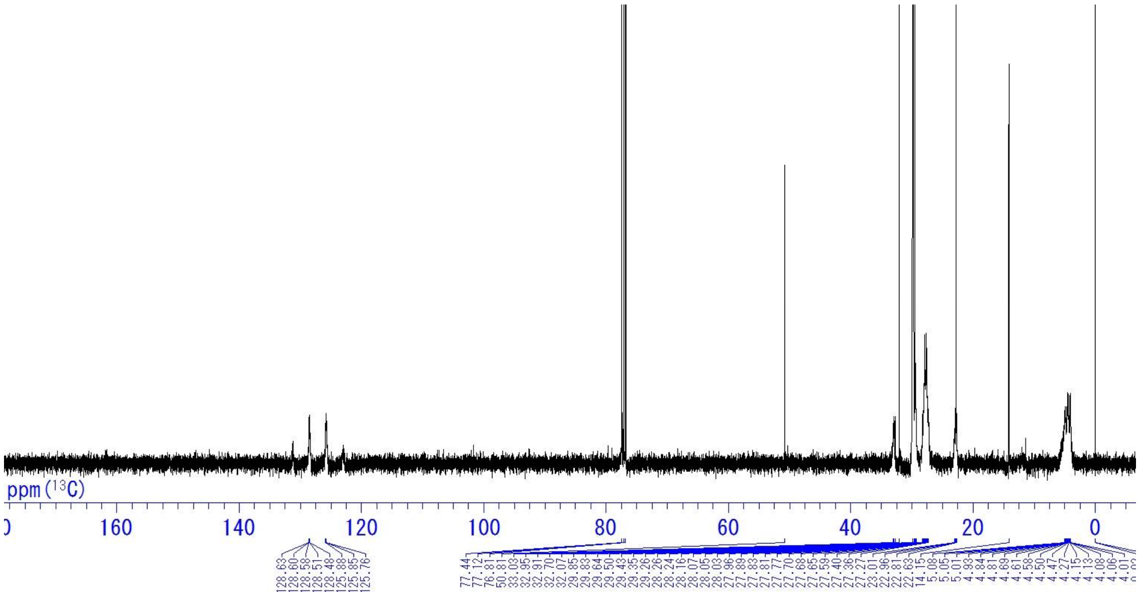

Chart S8. ^{13}C NMR spectrum of F+C18 POSS in $CDCl_3$.

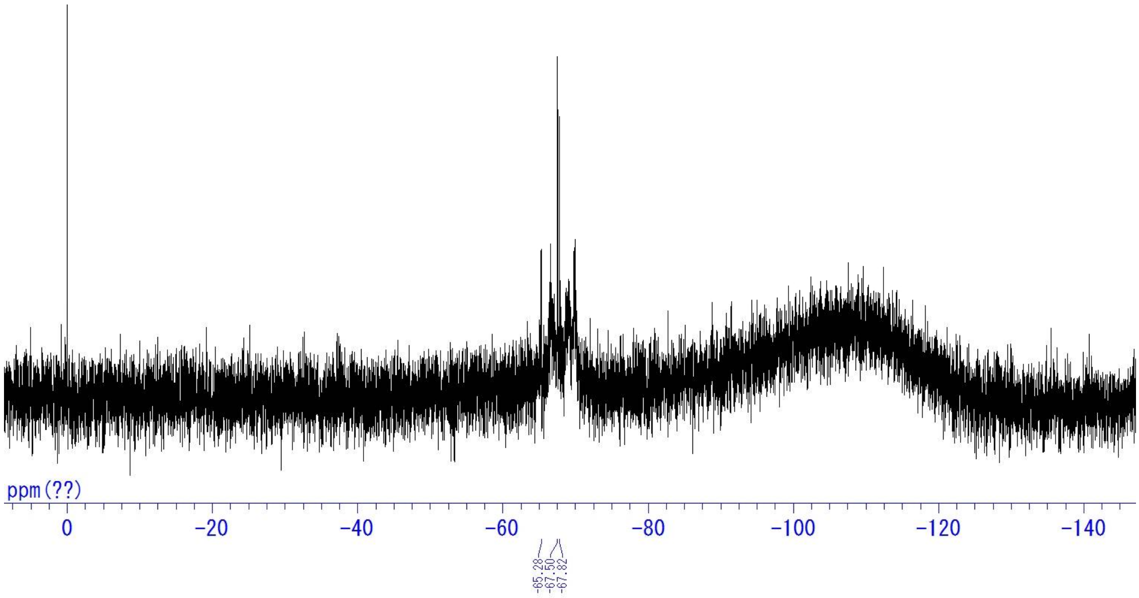

Chart S9. ^{29}Si NMR spectrum of F+C18 POSS in $CDCl_3$.

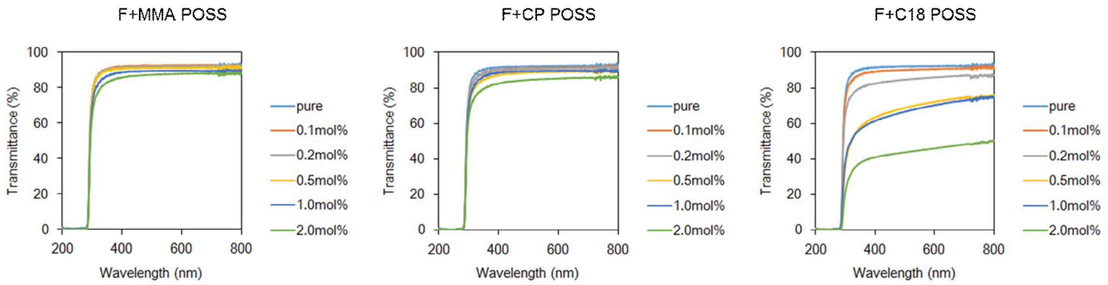

Figure S1. UV−vis transmittance spectra of PMMA hybrid films with variable concentrations of POSS fillers.

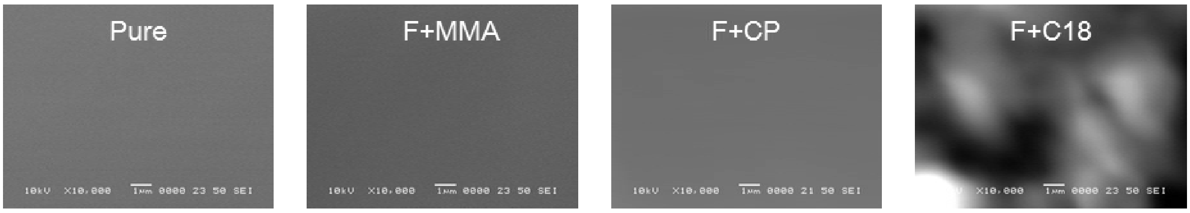

Figure S2 SEM images of PMMA hybrids containing POSS fillers (2 mol%). Scale bars represent 1 μm.

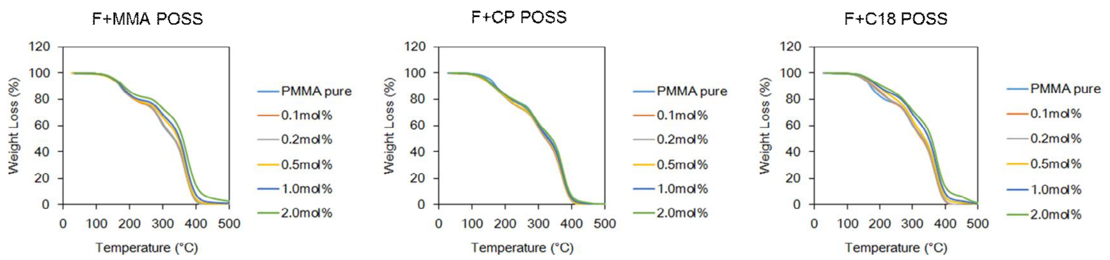

Figure S3. TGA thermograms of PMMA hybrids containing POSS fillers with a heating rate of 10 °C/min under nitrogen atmosphere.
